# Prognostic factors associated with visual outcome of salvageable eyes with posttraumatic endophthalmitis

**DOI:** 10.1038/s41598-019-49117-w

**Published:** 2019-09-03

**Authors:** Xuehui Lu, Honghe Xia, Chuang Jin, Weiqi Chen, Danny Siu-Chun Ng, Hua Yan, Haoyu Chen

**Affiliations:** 1 Joint Shantou International Eye Center, Shantou University & the Chinese University of Hong Kong, Shantou, China; 2 Department of Ophthalmology and Vision Sciences, Chinese University of Hong Kong, Hong Kong, China; 3 Department of Ophthalmology, Tianjin Medical University General Hospital, Tianjin, China

**Keywords:** Retinal diseases, Outcomes research

## Abstract

The purpose of this study is to evaluate the prognostic factors associated with visual outcomes in the salvageable eyes with posttraumatic endophthalmitis. We retrospectively reviewed the medical records of all patients diagnosed with posttraumatic endophthalmitis in our hospital between 2008 and 2015. The following information was collected: age, sex, etiology, past medical history, clinical manifestations, wound location, microbiology, blood leukocyte counts, types of interventions, initial visual acuities and final visual acuities. Univariate and multivariate analyses were used to explore the factors associated with final best-corrected visual acuity. In total, 98 eyes of 98 patients were included in our study. Fifty-seven eyes underwent vitrectomy, 27 of them had silicone oil tamponade, 38 eyes received intravitreal ceftazidime only and 3 eyes received intracameral ceftazidime. In univariate analysis, poor initial visual acuity, presence of intraocular foreign body, number of intravitreal injections, retinal detachment and Zone 3 injury were associated with poor visual outcome. In multivariable analysis, poor initial visual acuity, presence of intraocular foreign body and number of intravitreal injections were independently associated with poor visual outcome. The silicone oil group had fewer repeated intravitreal injections than the group without oil tamponade. We concluded that the visual outcome of salvageable eyes with posttraumatic endophthalmitis is associated with initial visual acuity, presence of intraocular foreign body and number of intravitreal antibiotic injections.

## Introduction

Endophthalmitis is a severe inflammatory disease that may be caused by trauma, intraocular surgery, infectious keratitis and endogenous causes. It leads to blindness in the absence of timely management. Posttraumatic endophthalmitis is a severe complication of ocular trauma and its management is more complex than other types of endophthalmitis. Li *et al*.^1^ reported a final visual acuity <20/200 in 60% of cases with posttraumatic endophthalmitis. Asencio *et al*.^2^ reported a case-control study of posttraumatic endophthalmitis and found that 73% of the eyes had a final visual acuity <20/200. It is certainly important to study the prognostic factors associated with visual outcome of posttraumatic endophthalmitis. Previous studies have reported that culture positivity, presence of virulent organisms, delayed treatment and retinal detachments were associated with poor visual outcomes in posttraumatic endophthalmitis^2–8^. However, whether baseline visual acuity, or the presence of intraocular foreign body affect visual outcome is controversial. While some reports found that baseline visual acuity did not influence the final visual outcome^5,8^, others reported that baseline visual acuity was significantly associated with visual outcomes of posttraumatic endophthalmitis^2,4,9^. There were few studies suggested that the presence of intraocular foreign body was not associated with final visual acuity of posttraumatic endophthalmitis^2,4,8^. However, Asencio *et al*.^2^ reported that the presence of intraocular foreign body was associated with worse final visual acuity.

Currently, the best available evidence for treatment of endophthalmitis comes from the Endophthalmitis Vitrectomy Study (EVS)^10^. The study reported that when initial visual acuity was better than hand movements, there was no significant difference in visual outcome for patients that received intravitreal antibiotics compared to those who received vitrectomy. If initial visual acuity was worse than light perception, the visual outcome for eyes that received vitrectomy was better than eyes that received intravitreal antibiotics. It is noteworthy that patients in EVS had post-cataract endophthalmitis, so the conclusions from EVS may not be directly applicable to other types of endophthalmitis. A few studies also compared different management options for posttraumatic endophthalmitis^11^. Our previous study has reported the factors associated with enucleation or evisceration for endophthalmitis^12^. In this study, we report the prognostic factors associated with visual outcomes in salvageable eyes with posttraumatic endophthalmitis.

## Methods

This study was approved by the Institutional Review Board of Joint Shantou International Eye Center of Shantou University and the Chinese University of Hong Kong. Informed consent was obtained from all participants or their parents if the patients are under 18 years old. The research followed the tenets of the Declaration of Helsinki. We retrospectively reviewed the medical records of patients who were diagnosed with posttraumatic endophthalmitis from our hospital between 2008 and 2015. Post- traumatic endophthalmitis was defined as traumatized eyes with pain, decreased visual acuity, photophobia and tearing, purulent exudate at the site of injury, eyelid edema, chemosis, hypopyon, fibrin in the anterior chamber and vitreous haze^5^. The following data was collected: age, sex, etiology, past medical history, clinical manifestation, wound location, microbiology, blood leukocyte counts and types of interventions, best-corrected visual acuity at presentation and last follow up. Patients with the following criteria were excluded: (A) underwent evisceration or enucleation; (B) follow-up duration <3 months; (C) clinically significant cataract which affected the visual acuity; (D) history of age-related macular degeneration, retinal detachment, glaucoma, or diabetic retinopathy.

According to the Ocular Trauma Classification Group^13^, the location of wound was classified into 3 Zones: Zone 1: cornea and limbus; Zone 2: limbus to 5 mm posterior into sclera; Zone 3: posterior to 3 mm from the limbus. Visual acuity measurement was performed using the Chinese standard logarithm visual chart, and the results were converted into Logarithm of minimal angle of resolution (LogMAR) unit. The arbitrary LogMAR values for visual acuity less than counting finger were used as follows: counting fingers was converted to 2.0 LogMAR units, hand motions was converted to 2.3 LogMAR units, light perception was converted to 2.5 LogMAR units, and no light perception was converted to 3.0 LogMAR units^14^.

The treatments for posttraumatic endophthalmitis were determined by the attending physicians, which included intravenous antibiotics, intravitreal injection of antibiotics (ceftazidime monotherapy), standard pars plana vitrectomy with or without silicone oil tamponade.

Spearman correlation analysis was used to examine the relationship between the final best-corrected visual acuity and age, interval from trauma to intervention, initial best-corrected visual acuity, blood leukocyte counts, days of intravenous antibiotics treatment, and the number of intravitreal antibiotics injection. Mann-Whitney test was used to analyze the relationship between final visual acuity and gender, with or without hypopyon, intraocular foreign body, retinal detachment, intravitreal antibiotics, vitrectomy with or without silicone oil tamponade. Kruskal-Wallis test was used to compare the visual outcomes between different wound locations. Finally, all the variables with p value < 0.1 in univariate analysis were included in multiple linear regression analysis. The level of significance was defined as p-value less than 0.05. All the statistical analyses were performed with SPSS software version 17.0 (SPSS Inc, Chicago, IL).

## Result

There were 142 eyes of 142 patients diagnosed with posttraumatic endophthalmitis at Joint Shantou International Eye Center of Shantou University and the Chinese University of Hong Kong between 2008 and 2015. Seven eyes that received evisceration or enucleation and 37 eyes with follow-up duration of less than 3 months were excluded. A total of 98 eyes of 98 patients were included in our study, consisting of 10 women and 88 men. Table 1 shows the characteristics of these patients. The mean age was 32.57 ± 17.78 (range from 4 to 73) years. There were thirty eyes (30.6%) with intraocular foreign body. Twenty-two eyes (22.4%) were injured in Zone 1, thirty-six eyes (36.7%) in Zone 2 and forty eyes (40.8%) in Zone 3. The mean interval from trauma to intervention was 3.79 ± 6.15 (range from 0.5 to 33) days. There were 52 eyes with hypopyon and 7 eyes with retinal detachment. The mean initial best corrected visual acuity was 2.02 ± 0.65 LogMAR units, and the mean final best-corrected visual acuity was 1.52 ± 1.17 LogMAR units. The mean blood leukocyte counts were 11.29 ± 3.68*10^9^/L. Table 2 shows the microbial culture results of the injured eyes and Table 3 shows the result of drug sensitivity test. The specimens were taken during the vitrectomy or before the intravitreal injections in the operation room. We used vitreous fluid as specimen in this study. We used nutrient broth as an inoculated media and a transport medium. When the specimen was taken into the microbiology lab, other kinds of medium including blood agar plate would be used to isolate different microorganism. 15 cases had positive microorganism culture in which 11 of them were gram positive, 4 cases were gram negative and 1 case contained both gram positive and gram negative microorganisms. *Staphylococcus epidermidis* was the most commonly isoloated microorganism. The rest of 81 cases had negative culture results. Of the 5 patients with intraocular foreign body with positive microbial culture, 4 were *Staphylocccus epidermidis* and 1 was *Bacillus subtilis*.

**Table 1:** Characteristics of the included patients with posttraumatic endophthalmitis.

Factors	Value
Age	32.57 ± 17.78 years
Gender	
Female	10 (10.1%)
Male	88 (89.8%)
Location of wound	
Zone 1	22 (22.4%)
Zone 2	36 (36.7%)
Zone 3	40 (40.8%)
Intraocular foreign body	30 (30.6%)
Interval from trauma to intervention	3.79 ± 6.15 days
Initial visual acuity	2.02 ± 0.65 (LogMAR)
Hypopyon	52 (53.1%)
Retinal detachment	7 (7.1%)
Microorganism culture	
Bacteria	16 (16.3%)
Fungus	1 (1.0%)
Blood leukocyte count	11.29 ± 3.68 (*10^9^/L)
Treatment	
Duration of intravenous antibiotics	6.52 ± 2.80 days
Vitrectomy	57 (58.2%)
With silicone oil tamponade	27 (27.6%)
Without silicone oil tamponade	30 (30.6%)
Intravitreal antibiotics	38 (38.7%)
Intracameral antibiotics	3 (3.1%)
The times of intravitreal antibiotics	0~7 times
Lens extraction	64 (65.3%)
Final visual acuity	1.54 ± 1.17 (LogMAR)

**Table 2:** Microbiology culture results

Samples	N.	Mean times of intravitreal antibiotics	Only intravitreal antibiotics	PPV	PPV + oil	Visual acuity (LogMAR)
Culture negative	81	0.91 ± 0.94	33	23	23	0.94 ± 1.00
Culture positive	16					
Gram negative	5	0.80 ± 1.30	1	2	1	1.24 ± 1.05
*Pseudomonas aeruginosa*	1	1	1	1		1.3
*Xanthomonas maltophilia pneumonia*	1	0	0	0	1	0.30
*Escherichia coli*	1	3	1	0	0	3.0
*Serratia marcescens*	1	0	0	1	0	0.70
*Burkholderia cepacia*	1	0	0	0	0	0.92
Gram positive	12	2.12 ± 1.8	4	4	1	1.67 ± 1.02
*Staphylococcus epidermidis*	6	0.82 ± 0.67	2	3	1	1.34 ± 1.00
*Staphylococcus aureus*	1	0	0	0	0	0.40
Viridans streptococci	2	5	0	0	0	2.5
*Bacillus subtilis*	1	1	1	0	0	2.3
*Staphylococcus capitis*	1	4	0	1	0	1.3
*Enterococcus gallinarum*	1	3	1	0	0	3

PPV: Pars plana vitrectomy.

**Table 3:** The results of drug susceptibility test for microorganism.

Microorganism	Cefazolin	Cefuroxime	Ceftazidime	Levofloxacin	Vancomycin	Ceftriaxone	Ciprofloxacin	Clindamycin
*Staphylococcus epidermidis*①	N	N	N	S	S	N	N	R
*Staphylococcus epidermidis*②	N	N	N	N	S	N	S	S
*Staphylococcus epidermidis*③	N	N	N	N	N	N	N	N
*Staphylococcus epidermidis*④	N	N	N	S	N	N	N	N
*Staphylococcus epidermidis*⑤	N	R	N	N	S	S	N	N
*Staphylococcus epidermidis*⑥	N	N	N	S	N	N	N	N
Viridans streptococci①	R	N	S	S	N	R	R	N
Viridans streptococci②	S	S	S	N	R	N	N	N
*Xanthomonas maltophilia pneumonia*	R	N	N	S	N	N	N	N
*Pseudomonas aeruginosa*	R	R	S	S	N	N	N	N
*Staphylococcus capitis*	N	N	N	S	S	N	S	N
*Burkholderia cepacia*	N	N	N	S	N	N	S	R
*Staphylococcus aureus*	S	N	S	N	S	N	S	R
*Serratia marcescens*	N	N	S	S	N	N	N	N
*Bacillus subtilis*	N	N	N	N	N	N	N	N
*Escherichia coli*	S	S	S	S	N	N	N	N
*Enterococcus gallinarum*	N	N	N	S	R	N	N	N

R: resistance; S: sensitive; N: not done.

All patients received intravenous antibiotics and wound repair immediately. Ceftazidime was the first choice empirical antibiotic, and was administered via both intravenous (1 g or 2 g bid) and intravitreal (2 mg/0.1 ml) routes, because of its broad spectrum effect. If patients had known allergic history to ceftazidime, intravenous (0.5 g qd) and intravitreal vancomycin (1 mg/0.1 ml) were prescribed 3 times in one patient. The mean duration of intravenous antibiotics was 6.52 ± 2.80 days. For patients with intraocular foreign bodies, vitrectomy with extirpation of foreign bodies would be performed. There were 57 eyes (58.2%) that received vitrectomy, 30 eyes without silicone oil tamponade and 27 eyes with silicone oil tamponade. 38 eyes received intravitreal antibiotics only. Intracameral antibiotics were used in 3 eyes. The indication of repeated intravitreal antibiotic injection was at the discretion of the attending physician based on clinical and B-scan ultrasonography examinations. It was prescribed at least 48 hours following the first injection. Among the 30 vitrectomized eyes without silicone oil temponade, 8 eyes had received one repeated injection of intravitreal antibiotic and one eye underwent revision vitrectomy followed by silicone oil tamponade. None of the 27 eyes with silicone oil tamponade received a second dose of intravitreal antibiotic. 62 eyes (63.3%) had significant cataract at first visit and underwent simultaneous lens extraction. One eye received lens extraction three months after the first treatment. One of the patients was initially treated with intravenous antibiotics, vitrectomy combined with intravitreal ceftazidime. However, these interventions failed to control the infection and progressed to keratitis. Subsequently, fungus was identified by HRT3 and the patient received intravenous fluconazole and intraocular lavage twice by fluconazole (3 mg/500 ml).

Table 4 shows the associations between final visual acuity and age, interval from trauma to intervention, initial visual acuity, blood leukocyte counts, duration of intravenous antibiotics, and number of intravitreal injections. Initial visual acuity was significantly associated with final visual acuity (r = 0.459, p < 0.001). Age was also significantly associated with final visual acuity (r = 0.233, p = 0.021). No significant associations were found between final visual acuity with interval from trauma to intervention, number of intravitreal antibiotics, blood leukocyte counts, or duration of intravenous antibiotics.

**Table 4:** Spearman correlation coefficient between the factors and the final visual acuity.

Factors	coefficient	P value
Age	0.233	0.021
Interval from trauma to intervention	0.159	0.117
Initial visual acuity	0.498	<0.001
Blood leukocyte count	0.135	0.184
Duration of intravenous antibiotics	0.106	0.300
The times of intravitreal antibiotics	0.174	0.086

Table 5 reveals the relationships between final visual acuity and gender, wound anatomy, hypopyon, intraocular foreign body, retinal detachment, vitrectomy with or without silicone oil tamponade, intravitreal antibiotics and positivity of microbiology culture. Mann-Whitney test revealed that retinal detachment resulted in reduced final visual acuity (p = 0.029) while gender, hypopyon did not affect final visual acuity. The final visual acuity of eyes with intraocular foreign body (1.34 ± 1.13 LogMAR) was worse than eyes without intraocular foreign body (0.91 ± 0.93 LogMAR). Kruskal-Wallis test showed that wound location significantly affected visual outcome (p = 0.031), and the final visual acuity difference between eyes with Zone 2 and Zone 3 injuries was significant (p = 0.011). The mean final visual acuities of vitrectomized eyes with silicone oil tamponade, vitrectomized eyes without silicone tamponade and non-vitrectomized eyes were: 0.88 ± 0.75 LogMAR, 0.98 ± 1.00 LogMAR, 1.18 ± 1.00 LogMAR. However, their differences were not statistically significant (p = 0.843).

**Table 5:** Comparison of final visual acuity between different groups.

Factors	VA (LogMAR)	P value
Gender		0.261*
Male	1.10 ± 1.04	
Female	0.61 ± 0.60	
Location of wound		0.031^#^
Zone 1	0.98 ± 1.06	
Zone 2	0.75 ± 0.78	
Zone 3	1.41 ± 1.12	
Intraocular foreign body		0.077*
With	1.34 ± 1.13	
Without	0.91 ± 0.93	
Hypopyon		0.633*
With	1.05 ± 1.09	
Without	1.03 ± 0.93	
Retinal detachment		0.029*
With	1.94 ± 1.18	
Without	0.97 ± 0.97	
Treatment		0.843^#^
Vitrectomy		
With silicone oil tamponade	0.88 ± 0.75	
Without silicone oil tamponade	0.98 ± 1.00	
Intravitreal antibiotics	1.18 ± 1.18	

^*^Mann-Whitney test; ^#^Kruskal-Wallis test; VA: Visual acuity.

Multiple linear regression analysis found that initial visual acuity (b = 0.652, p < 0.001), presence of intraocular foreign body (b = 0.462, p = 0.020) and increased number of intravitreal injections (b = 0.200, p = 0.013) were independently associated with final best-corrected visual acuity after confounding factors were adjusted (Table 6).

**Table 6:** Results of multiple linear regression analysis of associated factors with final visual acuity.

Factors	Beta coefficient	P value
Age	0.073	0.426
Initial visual acuity	0.652	<0.001
Intraocular foreign body	0.462	0.020
Location of wound		
Zone 1	0.031	0.728
Zone 2	−0.083	0.395
Zone 3	0.056	0.594
Retinal detachment	0.175	0.056
The times of intravitreal antibiotics	0.200	0.013

## Discussion

Our study found that poor initial visual acuity, number of intravitreal injection (ceftazidime monotherapy) and presence of intraocular foreign body were independent prognostic factors associated with visual outcome of salvageable eyes with posttraumatic endophthalmitis. We also found that vitrectomy with silicone oil tamponade was associated with a reduced number of intravitreal injections.

Brinton *et al*.^3^ retrospectively reviewed nineteen patients with posttraumatic endophthalmitis, and found that Gram-negative bacteria, retinal detachment and delayed treatment were related to poor visual outcome. Among vitrectomized eyes for posttraumatic endophthalmitis, Azad *et al*.^11^ reported 7 patients (58%) with silicone oil tamponade reached final visual acuity of >20/200 but only 1 patient (8.3%) without silicone oil tamponade could regain final visual acuity of >20/200. The authors suggested that silicone tamponade could be associated with better visual prognosis. Count *et al*.^4^ found that eyes with initial visual acuity better than light perception, negative culture for microorganism, and without retinal detachment were associated with good visual outcome in their prospective study of 17 patients with posttraumatic endophthalmitis. Asencio *et al*.^2^ retrospectively reviewed 15 patients with posttraumatic endophthalmitis and found that poor visual outcome was associated with intraocular foreign body, poor initial visual acuity and retinal detachment. Nicoara *et al*.^5^ reported that retinal detachment and delayed treatment resulted in poor visual outcome in their prospective study of 14 cases with posttraumatic endophthalmitis. Al-omran *et al*.^9^ retrospectively reviewed 67 patients with posttraumatic endophthalmitis, and found that virulent microorganisms, poor baseline visual acuity and delayed treatment were related to poor visual outcome. Thompson *et al*.^7^ isolated coagulase-negative staphylococcus from eleven patients with posttraumatic endophthalmitis and found 7 patients had final visual acuity equal to or better than 20/400. The authors proposed that virulence of microorganisms may be related to visual outcome. Jiang *et al*.^15^ retrospectively reviewed 121 eyes with traumatic endophthalmitis and suggested pars plana vitrectomy surgery as the preferred primary treatment. Our study of 98 eyes is considered to be one of the larger cohorts among previous studies of posttraumatic endophthalmitis, We have shown that baseline visual acuity, presence of intraocular foreign body and number of intravitreal injections were significantly associated with final visual outcome.

There are three possible reasons for greater number of intravitreal injections associate with poor visual outcome in our study. Firstly, it could be due to suboptimal control of a highly virulent microbial infection. For example, viridans streptococci was sensitive to vancomycin and cephalosporin (Table 3). However, at least 3 intravitreal antibiotics injections were required to control the infection. It is reported that viridans streptococci has cytotoxicity to retinal pigment epithelial cells^16^. One of our patients with polymicrobial infections received 3 intravitreal antibiotics injections. Hence, polymicrobial infections are likely to require higher number of intraocular injections. It has been reported that endophthalmitis resulted in severe retinal vasculitis, retinal vascular occlusion, hemorrhage, edema even dissolution of the retina^17,18^. Our previous study^19^ revealed that retinal vasculitis secondary to endophthalmitis led to atrophy of retinal inner layers and it was an independent risk factor associated with visual impairment. The second reason is the retinal toxicity from the intraocular antibiotics. There were several case reports that demonstrated the retinal toxicity of intraocular injection of amikacin, gentamicin and vancomycin^20–30^. Oum *et al*.^31^ found that frequent intravitreal antibiotics resulted in retinal toxicity in rabbit eyes and such toxicity was dose-dependent. Hence, frequent intravitreal antibiotics are not preferred when managing posttraumatic endophthalmitis. Third reason is the antibiotic resistance. It is well known that ceftazidime has fairly broad coverage but its gram positive coverage is inferior to that of vancomycin. However, the most common cause of traumatic endophthalmitis is gram positive organisms^32^. In our study, we used ceftazidime monotherapy as the first line therapy. As a result, the patients with gram positive organisms had a greater number of injections than the patients with gram negative organisms and culture negative organisms. This may be a possible resistance mechanism.

Our study found that none of the vitrectomized eyes with silicone oil tamponade required additional intravitreal antibiotic. Therefore, vitrectomy with silicone oil tamponade may reduce the need for subsequent intravitreal antibiotics. Several studies had suggested that vitrectomy facilitated the clearance of microorganism and inflammatory factors^33–36^. Some *in vitro* studies also reported that silicone oil had antimicrobial activity against *Staphylococcus aureus*, S*taphylococcus epidermidis*, *Pseudomonas aeruginosa*, *Candida albicans* and *Aspergillus*^37–40^. The susceptibility profile in our study showed that not all the microorganisms were sensitive to vancomycin and cephalosporin. One of our cases received vitrectomy combined with silicone oil tamponade and without any intravitreal antibiotics. The infection was controlled and resulted in good visual outcome.

Our study is limited by its retrospective design. A prospective study would validate the prognostic factors that predict outcomes in posttraumatic endophthalmitis. The microbial yield from our specimen culture was low in our study. It is possibly due to preoperative use of antibiotics and that the foreign bodies were not used for culture. Due to low culture positive rate, the relationship between the infecting microorganisms and the disease severity or the final visual acuity cannot be convincingly established. Advanced techniques such as polymerase chain reaction (PCR) may help to improve microbial yield from future specimens. For eyes that received multiple intravitreal injections of antibiotics, poor outcomes can be attributed to highly virulent infection, prolonged disease course, drug toxicity as well as drug resistance. Ceftazidime and vancomycin resistance patterns were not characterized for all isolates.

In conclusion, our study found that initial visual acuity, presence of intraocular foreign body and numbers of intravitreal injections were significantly associated with poor visual outcome in salvageable eyes with posttraumatic endophthalmitis. In our opinion, if there was difficulty to perform vitrectomy, intravitreal antibiotics could still be an effective treatment for posttraumatic endophthalmitis. Early indication for vitrectomy should be considered when the infection has not been controlled after initial intravitreal injection of antibiotics. In the presence of intraocular foreign body or retinal detachment, primary surgical treatment may lead to better outcome. Vitrectomy with silicone oil tamponade may also assist in controlling posttraumatic endophthalmitis. Further studies to confirm the results are warranted.
